# A Review of Gel-Based Materials for Electromagnetic Devices

**DOI:** 10.3390/gels12070600

**Published:** 2026-07-06

**Authors:** Lei Huang, Hongrui Xu, Yizhou Zhang, Haoyang Zhang

**Affiliations:** 1 Institute of Flexible Brain-Computer Interface, School of Chemistry and Materials Science, Nanjing University of Information Science and Technology, Nanjing 210044, China; kyo_qq@hotmail.com (L.H.); yizhou.zhang@nuist.edu.cn (Y.Z.); 2 School of Electronic and Optical Engineering, Nanjing University of Science and Technology, Nanjing 210094, China; xhr@njust.edu.cn; 3 School of Electronics and Information Engineering, Nanjing University of Information Science and Technology, Nanjing 210044, China

**Keywords:** hydrogel, aerogel, ionogel, electromagnetic devices, electromagnetic wave absorption, antenna, EMI shielding, radome, flexibility, lightweight materials

## Abstract

Gel-based materials are emerging as lightweight, mechanically compliant, and electromagnetically tunable platforms for next-generation antennas, electromagnetic interference (EMI) shields, microwave absorbers, and radomes. This review summarizes recent progress in hydrogel-, aerogel-, ionogel-, organohydrogel-, and xerogel-based electromagnetic materials, with emphasis on how network structure, pore architecture, solvent phase, and functional fillers regulate permittivity, conductivity, impedance matching, and attenuation. The device-level roles of gels are discussed in miniaturized and reconfigurable antennas, absorption-dominated shielding systems, broadband microwave absorbers, high-temperature wave-transparent radomes, and metamaterial, energy-harvesting, and bioelectronic systems. Particular attention is paid to the mechanisms of dipolar relaxation, ionic conduction, interfacial polarization, conduction loss, magnetic loss, and multiple scattering. Finally, key challenges are identified, including hydrogel dehydration and freezing, aerogel fragility, ionogel cost and leakage, limited long-term reliability, and the lack of standardized performance metrics. Future directions toward durable, scalable, multifunctional, and device-integrated gel-based electromagnetic materials are proposed.

## 1. Introduction

Emerging technologies such as wearable electronics, soft robotics, sixth-generation (6G) communication, and intelligent aerospace systems are driving new requirements for electromagnetic (EM) materials. Beyond electromagnetic performance, these systems increasingly demand mechanical flexibility, lightweight design, conformability, and long-term environmental stability [1,2,3]. Achieving these properties simultaneously remains challenging. Conventional EM materials typically involve clear trade-offs. Metals and ceramics provide excellent conductivity and thermal stability but are rigid and difficult to integrate with curved or deformable structures [4,5]. In contrast, flexible polymers and elastomers offer mechanical compliance but often exhibit limited electromagnetic tunability or reduced performance at high frequencies. This trade-off has become a key bottleneck as EM systems evolve from rigid planar architectures toward conformal and functional devices.

Gel-based materials serve as a flexible platform for next-generation electromagnetic devices. Gels are crosslinked polymer networks that confine large amounts of liquid or gas within their structure. Depending on the internal phase, they can be categorized as hydrogels, aerogels, ionogels, organohydrogels, and xerogels [6,7,8]. In fact, the composition and porosity of these gels can be continuously tuned. These two structural parameters together control both the mechanical and electromagnetic behavior. Because of this structural tunability, the electromagnetic properties become highly adjustable. For example, the permittivity can span orders of magnitude, and various loss mechanisms can be introduced by tailoring the composition and pore architecture. These properties are systematically examined in Section 2. From a device perspective, gels offer several advantages. Their softness enables conformal integration with curved and dynamic surfaces [9,10]. Porous architectures such as aerogels improve impedance matching and facilitate wave penetration into loss-active regions [11,12]. In many cases, dielectric and conductive losses coexist within a single structure, reducing the need for complex multilayer designs [13].

Driven by these advantages, gels have been employed across antennas, EMI shielding, microwave absorption, radomes, and emerging areas including terahertz (THz) devices, metasurfaces, energy harvesting, soft actuators, and biointerfaces [11,14,15,16,17,18,19,20,21,22,23,24,25,26,27]. Representative advances in each application domain are surveyed in Section 3.

Despite these advances, significant material-level challenges persist, including environmental stability, mechanical reliability, and scalable manufacturing. These challenges and future research directions are discussed in Section 4. The central challenge of gel-based electromagnetic materials is to resolve the inherent coupling between electromagnetic functionality, mechanical deformability, and environmental stability within a single material system.

In recent years, research on gel-based electromagnetic materials has grown rapidly. However, this rapid expansion has produced a fragmented research landscape, making it difficult to identify the common relationships between gel structure and electromagnetic performance. In addition, most existing reviews are organized either by material type or by application domain, which separates closely related concepts and limits cross-comparison between different systems.

To address this issue, this work provides a unified structure–property–function framework for gel-based electromagnetic materials. Unlike previous reviews that focus on a single material system or a specific application, we systematically compare gels across antennas, shielding, absorption, radomes, and emerging EM systems within a single perspective. This framework links microscopic gel architecture with macroscopic device behavior and highlights the key trade-offs among electromagnetic performance, mechanical flexibility, processing scalability, and long-term stability. By integrating these viewpoints, this review aims to provide a more coherent understanding of gel-based EM materials and to offer general design guidance for future material development and device engineering.

## 2. Fundamentals of Gels for Electromagnetic Devices

The electromagnetic performance of a gel is not determined by any single structural feature but by the combined effect of its network chemistry, pore architecture, and confined phase composition. This section examines these relationships in sequence. The key electromagnetic-related properties of gels are discussed first, followed by their structural classifications and the corresponding structure–property relationships. The underlying electromagnetic interaction mechanisms are then analyzed, and finally the application principles of gels in representative electromagnetic devices are briefly introduced as a transition to the detailed discussions in Section 3.

### 2.1. Properties of Gels in Electromagnetic Devices

**Figure 1:**
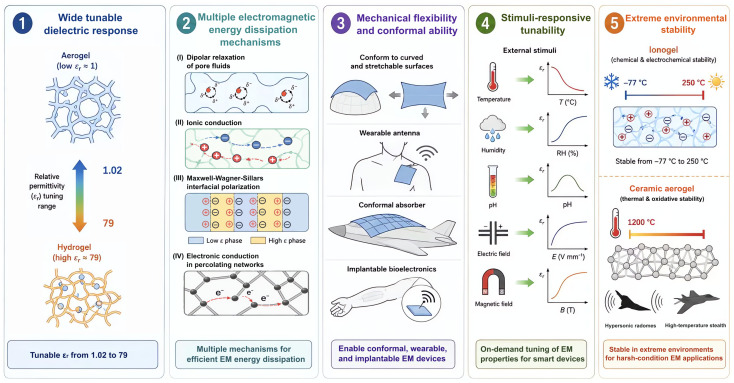
Key properties of gel materials for electromagnetic device applications. (**1**) Tunable dielectric response spanning a wide permittivity range from near-unity aerogels to high-permittivity hydrogels. (**2**) Multiple energy dissipation mechanisms including dipolar relaxation, ionic conduction, interfacial polarization, and electronic conduction loss. (**3**) Mechanical flexibility enabling conformal integration with curved and dynamic surfaces. (**4**) Stimuli-responsiveness allowing external tuning of electromagnetic behavior via temperature, humidity, pH, or electric and magnetic fields. (**5**) Tailorable environmental stability ranging from anti-freezing organohydrogels to ceramic aerogels stable above 1200 °C.

Compared with conventional rigid materials such as ceramics, metals, and dense polymers, gels have several unique properties that give them distinct advantages for electromagnetic devices (Figure 1).

First is their wide tunable dielectric response. Gel materials exhibit a wide range of relative permittivity $\varepsilon_{r}$, spanning from near unity ($\varepsilon_{r}\approx 1.02$–1.06) in porous ceramic aerogels [15] to values close to bulk water ($\varepsilon_{r}\approx 79$) in fully hydrated hydrogels [16]. Within each material system, $\varepsilon_{r}$ can be further tailored by modifying chemical composition, crosslinking density, and pore structure. This wide tunability can match the different permittivity requirements of electromagnetic devices. Complementing the dielectric tunability, gel density spans an exceptionally wide range from approximately 0.015 g·cm^−3^ in ultralight aerogels [19] to roughly 1 g·cm^−3^ in fully hydrated hydrogels, while specific surface areas reach 100–1000 m^2^ · g^−1^ in aerogel and cryogel systems [11,19]. Second is their diverse energy dissipation capabilities. Gels dissipate electromagnetic energy through multiple coexisting mechanisms including dipolar relaxation, ionic conduction, interfacial polarization, and electronic conduction, as analyzed in detail in Section 2.3. Salt-containing hydrogels typically exhibit ionic conductivities of $10^{-3}$ to $10^{-1}$ S·cm^−1^ and ionogels $10^{-4}$ to $10^{-2}$ S·cm^−1^ depending on ion concentration and mobility [13,28,29]. Third, most gels are mechanically flexible, conforming to curved or moving surfaces without losing electromagnetic function, which enables wearable antennas, conformal absorbers, and implantable bioelectronics that are difficult to achieve with rigid materials [27]. Fourth, gels are inherently tunable by external stimuli. Their electromagnetic response (e.g., resonance frequency or impedance) shifts directly with external conditions such as temperature, humidity, pH, and electric or magnetic fields. The gel therefore acts as a tunable component and enables self-tuning devices with simpler architectures [30]. Finally, the environmental stability of gels can be tailored for extreme conditions. Ionogels with non-volatile ionic liquids have been shown to remain stable from −77 °C to 250 °C, resisting evaporation and freezing over this range [25]. At the other extreme, certain ceramic aerogels can withstand over 1200 °C in air, making them suitable for hypersonic radomes and high-temperature stealth [15]. Together, these features give gels unique advantages over traditional electromagnetic materials, forming the foundation for diverse device applications.

### 2.2. Structure of Gels

**Figure 2:**
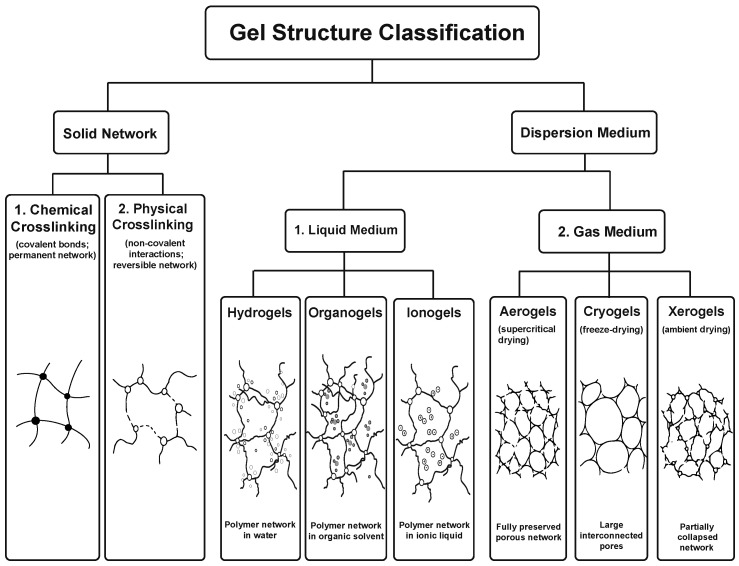
Schematic illustration of gel structure classification based on solid network crosslinking type and dispersion medium.

The two fundamental structural descriptors of a gel are the continuous solid network and the dispersion medium occupying the interstitial space, as illustrated in Figure 2.

The solid network is defined by its crosslinking architecture. Chemically crosslinked gels are linked by irreversible covalent bonds, which create a permanently locked network that restricts chain motion and stabilizes dielectric properties. Physically crosslinked gels rely on reversible non-covalent interactions. They enable environmental responsiveness but generally offer lower mechanical stability than their chemical counterparts [30].

The dispersion medium that fills the network interstices can be broadly divided into liquids and gases. Among liquid media, hydrogels contain a large amount of water, which contributes to their high permittivity and, in the case of ionic hydrogels, supports ion transport for conductivity [31]. Organogels and organohydrogels employ organic or organic/water binary solvents that suppress ice crystallization and retard evaporation. This ensures stable performance at subzero temperatures [32,33]. Ionogels immobilize ionic liquids within the network, eliminating solvent evaporation while retaining high ionic conductivity, a feature crucial for conductive and capacitive elements [23,34]. When the liquid medium is removed, the interstices are filled by gas, and the specific drying protocol dictates the final porous architecture. Aerogels, obtained by supercritical drying, preserve the wet network almost intact, featuring high porosity and low permittivity that favor impedance matching [35]. Cryogels (sometimes referred to as cryo-aerogels) are produced by freeze-drying, where the frozen solvent sublimates, creating a macroporous structure that is especially attractive for absorption-dominant EMI shielding [36]. Xerogels, formed by simple ambient-pressure drying, undergo substantial network collapse, trading ultra-high porosity for scalable manufacturing and mechanical robustness [37].

The pore architecture, defined by the solid network and the chosen filling and drying method, directly affects the electromagnetic performance. When the pore size is much smaller than the wavelength, the gel behaves as an effective medium with tailored permittivity. Graded porosity improves impedance matching, a principle widely used in low-loss radomes [38].

### 2.3. Mechanisms of Gels in Electromagnetic Interactions

The electromagnetic response of gel-based materials involves several distinct physical mechanisms that operate across different structural scales, from molecular-level dipolar relaxation to macroscopic multiple scattering. A systematic understanding of these mechanisms is essential for rational material design, as each mechanism can be independently tuned through gel composition and architecture.

Dipolar relaxation of polar species within the gel matrix provides energy dissipation at the molecular scale. In hydrogels, water molecules reorient under an alternating electromagnetic field and convert electromagnetic energy into heat through dielectric relaxation [13,39]. In organohydrogels, the binary organic/water solvent system introduces additional dipolar species and broadens the effective frequency range of this relaxation process [40]. The strength of dipolar loss depends on the concentration and rotational mobility of the polar species, both of which can be controlled through solvent composition and crosslinking density.

Ionic conduction provides a second, compositionally tunable loss channel. Mobile cations and anions in salt-containing hydrogels migrate under the oscillating electromagnetic field and generate Joule heating through ionic current [13,28]. Ionogels extend this mechanism to non-volatile ionic liquids, which maintain stable ionic conductivity over wide temperature ranges without the evaporation limitations of aqueous systems [29,41]. The ionic conduction loss scales with ionic mobility and concentration, and can be regulated by selecting ionic liquid species with appropriate viscosity and dissociation constants.

At the mesoscopic scale, interfacial polarization occurs at boundaries between phases with differing permittivity or conductivity. In filled gel systems, charge carriers accumulate at filler/matrix and filler/filler interfaces under an alternating electric field, forming distributed micro-capacitor structures that dissipate energy through Debye-type relaxation [19,42]. The magnitude of interfacial polarization loss is governed by the density and dielectric contrast of these internal interfaces, making it sensitive to filler loading, dispersion quality, and the intrinsic permittivity difference between the gel matrix and the embedded fillers.

Electronic conduction loss operates when conductive fillers establish percolating pathways within the gel. Electrons migrating through interconnected conductive channels dissipate electromagnetic energy resistively [11,12]. This mechanism is especially relevant for aerogel shields and absorbers incorporating carbon nanotubes, graphene, or MXenes. Maintaining moderate conductivity is critical, because excessive conductivity increases front-surface reflectivity and degrades impedance matching [43].

Magnetic loss mechanisms become active when magnetic fillers such as Fe_3_O_4_, CoNi, or ferrites are embedded in the gel. Natural ferromagnetic resonance, eddy current loss, and magnetic hysteresis supplement the dielectric loss pathways to broaden the effective absorption bandwidth [39,42]. Magnetic fillers also introduce the complex permeability $\mu_{r}=\mu^{\prime }-j\mu^{\prime \prime }$ as an additional design parameter for impedance engineering.

Beyond these intrinsic dissipation processes, the porous architecture of gels enables multiple scattering of incident electromagnetic waves. In aerogels and cryogels, interconnected pore networks cause repeated reflections and refractions at pore walls. These reflections and refractions extend the effective propagation path within the loss-active material and increase the probability of energy dissipation [11,19]. When the pore size is considerably smaller than the incident wavelength, the porous gel behaves as an effective medium whose apparent permittivity decreases systematically with increasing porosity, a principle widely exploited for impedance matching in radomes and lightweight absorbers [15,38].

All these mechanisms must be balanced through proper impedance matching to achieve efficient device-level performance. Impedance matching requires the characteristic impedance of the material to approach that of free space ($Z_{0}\approx 377\;\Omega$), thereby allowing electromagnetic waves to penetrate the material interior with minimal front-surface reflection [3,44]. This impedance condition is typically satisfied by balancing the complex permittivity and permeability, engineering porosity or composition gradients, and optimizing the material thickness relative to the operating wavelength. In gel systems, the independent tunability of network architecture, solvent composition, and filler distribution enables simultaneous impedance matching and strong internal attenuation across diverse electromagnetic applications. These mechanisms are activated and regulated by functional fillers embedded within the gel matrix. Table 1 summarizes the major filler categories used in gel-based electromagnetic materials, their representative compositions, and the electromagnetic properties they primarily enhance.

### 2.4. Application of Gels in Electromagnetic Devices

The properties, structures, and mechanisms described above map onto a broad spectrum of EM device functions, spanning antennas, EMI shielding, microwave absorption, radomes, and emerging areas such as metasurfaces and bioelectronic interfaces. These applications are surveyed in detail in Section 3. Table 2 provides a systematic cross-comparison of the five primary gel families across their key electromagnetic parameters, optimal device applications, and inherent limitations.

## 3. Gels in Electromagnetic Devices

Gel materials enable electromagnetic device applications via tailorable electromagnetic properties and stimuli-responsiveness. Over the past decade, extensive research has been conducted on gel-based electromagnetic devices. This section reviews representative advances of gels in antennas, electromagnetic shielding, microwave absorption, radomes, and other electromagnetic systems.

### 3.1. Gels in Antennas

An antenna converts guided electromagnetic waves to free-space radiation and vice versa, serving as a core component in wireless communications, radar, RFID, biomedical telemetry, and aerospace systems. The growth of wearable electronics, flexible IoT devices, and implantable medical systems demands lightweight, conformal, multifunctional antennas.

Conventional antennas, fabricated from rigid metal conductors on stiff dielectric substrates, struggle to meet these requirements. First, they have poor mechanical compliance: unstructured metal films often crack at tensile strains as low as 2%. This makes them unsuitable for dynamic curved surfaces in wearable and implantable devices [51,52]. Second, they have limited miniaturization potential: common low-$\varepsilon_{r}$ substrates ($\varepsilon_{r}\approx 2$–4) lead to bulky antenna footprints at sub-GHz frequencies, as antenna physical size is proportional to the medium wavelength, which decreases with rising $\varepsilon_{r}$ [16]. Finally, they lack integrated multi-functionality: frequency tuning relies on power-hungry PIN diodes. The rigid architecture cannot achieve intrinsic self-healing, biocompatibility, and optical transparency in one design [1,2,53]. These limitations have driven extensive research into gel-based materials as alternatives for future antenna design.

**Figure 3:**
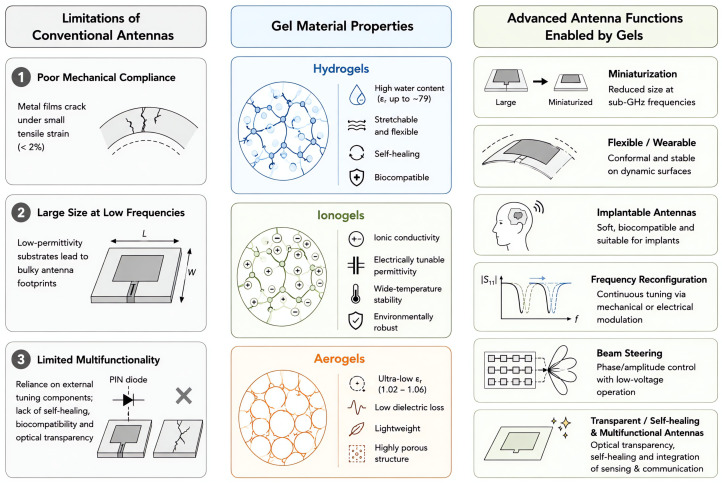
Gel materials for antenna applications.

Gels directly address these limitations via tailorable electrical and mechanical properties, as shown in Figure 3. Hydrogels with high water content, with reported values up to ≥95 wt% and $\varepsilon_{r}\approx 79$, enable antenna miniaturization at sub-GHz frequencies [16], while most hydrogel and ionogel systems are intrinsically stretchable and self-healing [9,10]. Aerogels with ultra-low $\varepsilon_{r}$ (1.02–1.06, near air) reduce dielectric loss to improve antenna bandwidth and gain [14,15], and ionogels offer wide temperature stability and electrically tunable dielectric properties for frequency-reconfigurable designs, avoiding the dehydration and freezing issues of hydrogels [54]. Gels can also be engineered for biocompatibility, optical transparency, and stimuli responsiveness, enabling monolithic integration of sensing and communication functions [55,56,57]. We systematically review recent advances in gel-based antennas in the following sections, organized by typical gel material systems.

#### 3.1.1. Aerogel-Based Antennas

Aerogel-based antennas exploit ultra-low dielectric constant, near-air density, and high thermal stability to achieve lightweight, low-loss antenna designs that overcome the weight and loss limitations of conventional rigid substrates.

**Figure 4:**
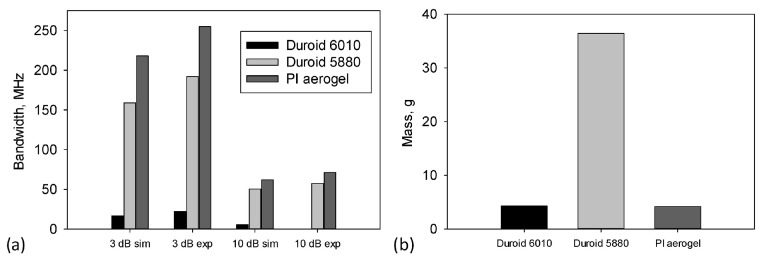
Comparison of (**a**) bandwidth (experimental and simulated), and (**b**) mass of antennas fabricated from polyimide aerogel (100% DMBZ, BPDA, and TAB), and Rogers Duroid 6010 and 5880. (Reprinted with permission from Ref. [14]. Copyright 2012 American Chemical Society).

For lightweight and low-loss antenna design, aerogels function either as the primary dielectric substrate or as a filler material. As a substrate, Meador et al. [14] developed polyimide (PI) aerogels. They fabricated planar patch antennas that achieved wider bandwidth, comparable or higher gain, and reduced weight versus identical commercial substrate antennas [14]. The relative dielectric constant of the aerogel varied linearly with its density, following a trend similar to that of silica aerogels. The most hydrophobic formulations, based on dimethylbenzidine (DMBZ) and biphenyltetracarboxylic dianhydride (BPDA) cross-linked with triaminophenoxybenzene (TAB), exhibited the lowest loss tangents and a relative permittivity as low as 1.16. A direct performance comparison is presented in Figure 4. Based on this work, Miranda et al. further demonstrated a 5 GHz 2×4 slot-coupled aerogel phased array with good scalability and link performance [58]. The low dielectric constant and low density of the polyimide aerogel enabled antennas with higher gain, wider bandwidth, and lower mass compared to conventional microwave laminates such as RT/Duroid. As a low-loss filler, Rodríguez-Solís et al. designed a slotted waveguide array loaded with PI aerogel, reporting 9 dBi gain, insertion loss below 0.15 dB, and one order of magnitude lower weight than commercial metal devices [59]. Abdel-Rahman et al. [60] fabricated a 7.2 GHz C-band patch antenna using polymer-encapsulated SiO_2_ aerogel substrates with a density of 0.123 g/cm^3^ and a porosity of 95%. The substrate exhibited a relative permittivity between 1.055 and 1.25 and a loss tangent ranging from $5.08\times 10^{-4}$ to 0.0206. The antenna achieved 1.5 dB higher gain, 88% wider bandwidth, and 68.5% lower weight than a Rogers RT5880 reference device [60].

For extreme or specialized environments, aerogels provide thermal stability or biocompatibility while maintaining radiation performance. For hypersonic vehicle applications, Chen et al. reported a composite dielectric resonant antenna using a high-temperature aerogel as both thermal insulation layer and dielectric loading component [61]. The aerogel had a relative permittivity of 1.38 and could withstand temperatures up to 1000 °C. The device achieved 4.8–10.4 GHz ultra-wideband response, 9.2 dBi peak gain, and continuous operation for 1280 s at 1000 °C, integrating radiation and thermal protection in a single gel-based device. For biomedical applications, Adnan et al. [62] fabricated a 2.4 GHz patch antenna on a biodegradable sago-starch aerogel substrate. It delivered favorable return loss, gain, and input impedance matching, with performance comparable to conventional FR-4 antennas for temporary implantable and body-worn devices [62].

#### 3.1.2. Hydrogel-Based Antennas

Unlike low-permittivity aerogel substrates that prioritize bandwidth and weight reduction, hydrogel-based antennas use their high water content for antenna miniaturization and mechanical reconfigurability, offering high permittivity, intrinsic stretchability, and stimuli responsiveness for frequency tuning and integrated sensing.

**Figure 5:**
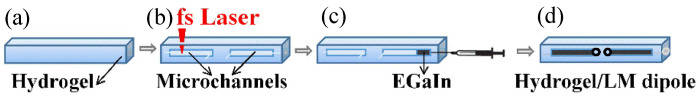
Fabrication procedure of the hydrogel/liquid metal dipole antenna. (**a**) Raw hydrogel substrate. (**b**) Femtosecond laser ablation to carve microchannels inside hydrogel. (**c**) Injecting liquid metal EGaIn into prefabricated microchannels. (**d**) Final integrated flexible hydrogel/LM dipole antenna (Adapted from Ref. [16]).

For miniaturized flexible antenna devices, the high dielectric constant of hydrogel substrates provides a material-level solution to the bulky footprint of conventional sub-GHz antennas. As shown in Figure 5, Zhao et al. fabricated a stretchable dipole antenna using femtosecond laser-patterned microchannels in a polyacrylamide (PAAm) hydrogel substrate filled with EGaIn liquid metal [16]. The PAAm hydrogel contained approximately 95 wt% deionized water and exhibited a high relative permittivity of 79 in the 0.5–1.5 GHz range. The device achieved $S_{11}=-12.6$ dB at 927.5 MHz, with the high permittivity reducing the dipole length by approximately 50% compared to conventional polymer substrates. Beyond size reduction, the hydrogel’s stretchable matrix enabled passive mechanical frequency reconfiguration, with resonant frequency shifting continuously from 770.3 MHz to 927.0 MHz as tensile strain decreased from 48% to 0%.

For integrated sensing antenna devices, the stimuli-responsive swelling of hydrogels enables passive, battery-free signal transduction, expanding antenna functionality beyond single radiation performance. Kim et al. integrated a pH-sensitive hydrogel with a 5 mm slot antenna to construct a passive wireless pH sensor [55]. In this design, a back-metal layer was coated directly on the pH-sensitive hydrogel. Swelling and deswelling of the hydrogel under different pH conditions altered the effective dielectric constant of the slot, producing a frequency shift of approximately 160 MHz across pH 5 to 12 at 21.7 GHz. Building on this design, Dautta et al. reported an RF/near-field communication (NFC) resonator antenna using a multifunctional hydrogel as the core functional interlayer for multi-parameter biosensing, with direct smartphone readout and a demonstrated hydrogel-based electronic skin device [56].

Targeted fabrication strategies have been developed to match hydrogel material characteristics, addressing processing barriers for practical antenna device applications. Sun et al. fabricated NFC/RFID antennas via DLP 3D printing of silver-loaded photocurable hydrogel ink [46], with post-printing dehydration forming percolating conductive networks in the hydrogel matrix (387 S/cm conductivity). The encapsulated hydrogel antenna maintained stable performance after 100% strain cycling and 30 days of ambient storage, validated for eye-movement recognition applications.

For performance optimization and low-cost applications, Cui et al. proposed a neural-network-based optimization method for flexible hydrogel antennas, achieving a 1.5-fold increase in RSS and 78% longer sensing distance [63]. Sen et al. demonstrated a low-cost ultra-high-frequency (UHF) RFID wetness sensor using a superabsorbent hydrogel as the core moisture-sensitive layer, with 1 m maximum read range and stable performance under bending [64].

For practical deployment, hydrogel antennas require effective encapsulation strategies to maintain stable dielectric and mechanical performance under varying ambient humidity and temperature, because antenna resonant frequency and impedance are directly coupled to the water content of the hydrogel matrix.

#### 3.1.3. Ionogel-Based Antennas

Ionogels are promising for reconfigurable and mechanically adaptive antennas due to their high ionic conductivity, large interfacial capacitance, flexibility, and environmental stability.

Ionogels can act as high-capacitance gate dielectrics in printed transistors for low-voltage operation and integration in flexible reconfigurable antennas. Grubb et al. [65] demonstrated a fully printed X-band phased-array antenna on flexible Kapton, using an ionogel gate dielectric in carbon nanotube (CNT) FETs. With a specific capacitance of 10 μF/cm^2^ and operating voltage below 3 V, the transistors reliably controlled phase shifters, enabling beam steering from 0° to 22.15° at 10 GHz [65].

Ionogels can also serve as mechanically tunable dielectric layers in flexible microstrip antennas for passive frequency reconfiguration via compression-induced thickness change. Zou et al. [66] proposed a multilayer antenna with an ionogel compressible core between polydimethylsiloxane (PDMS) layers. By tuning strain, it achieved dual-band tuning from 3.69–4.23 GHz (S-band) and 7.0–7.17 GHz (C-band), with reflection coefficients below −30 dB. The ionogel ensures stable performance under repeated bending [66].

Ionogels can further act as voltage-tunable dielectric patches in transmission-line gaps for continuous frequency reconfiguration. Zhou et al. reported a reconfigurable antenna in which the feed lines were connected via ionogel patches [67]. When a regulating voltage was applied across the ionogel, a capacitive structure formed between the feed lines. Electrons migrating along one feed line were deflected by this capacitive effect, reducing the current transferred to the other feed line and shifting the operating frequency. Under 0–5.5 V bias, the operating frequency shifted by approximately 0.1 GHz, and the tuning compensated bending-induced drift, enabling compact multi-band flexible designs.

Table 3 summarizes representative gel-based antennas categorized by the three core gel systems discussed in this work.

In summary, aerogel substrates excel in high-frequency, high-gain applications where low dielectric loss and ultralight weight are priorities, delivering bandwidth improvements up to 88% and weight reductions exceeding 68% versus commercial alternatives. Hydrogel substrates are most effective for antenna miniaturization and mechanical reconfigurability owing to their high permittivity and intrinsic stretchability, with demonstrated size reductions of approximately 50% relative to polymer substrates. Ionogel-based designs enable voltage-controlled frequency reconfiguration and wide-temperature operation without the evaporation limitations of hydrogels. The unresolved challenge common to all three approaches is environmental durability: aerogels need mechanical reinforcement against brittleness, hydrogels require encapsulation against dehydration and freezing, and the long-term reliability of ionogel antenna systems under repeated electrical cycling has yet to be systematically evaluated.

### 3.2. Gels in Electromagnetic Interference Shielding

**Figure 6:**
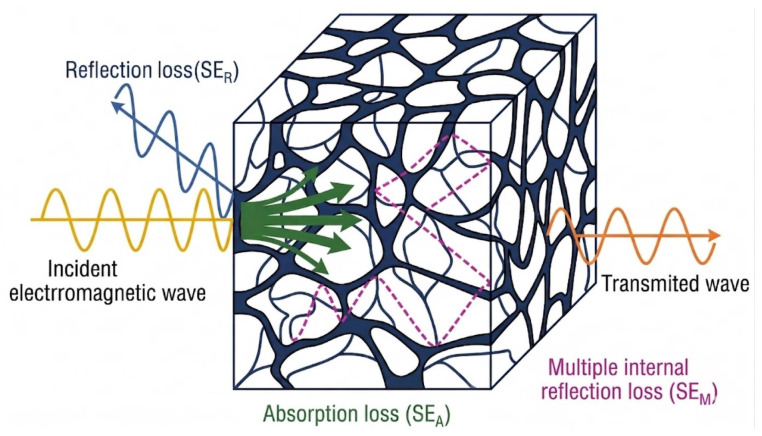
Three fundamental EMI shielding mechanisms: reflection loss ($SE_{R}$), absorption loss ($SE_{A}$), and multiple internal reflection loss ($SE_{M}$).

EMI shielding refers to the attenuation of incident electromagnetic waves through reflection loss ($SE_{R}$), absorption loss ($SE_{A}$), and multiple internal reflection loss ($SE_{M}$), as shown in Figure 6. The total shielding effectiveness ($SE_{T}$, in dB) is the sum of these three components, expressed as $SE_{T}=SE_{R}+SE_{A}+SE_{M}$ [68]. When $SE_{T}\geq 15$ dB, $SE_{M}$ is usually very small and can be ignored because most waves are already attenuated inside the material. Therefore, maximizing the $SE_{A}$ contribution provides a direct and effective design rule for achieving lightweight, absorption-dominated gel shields.

Current EMI shielding solutions face several limitations. Metallic shields such as copper plates, aluminum foils, and nickel-coated fabrics provide high shielding effectiveness (SE). However, their high density, poor conformability, and susceptibility to corrosion remain critical drawbacks for aerospace, wearable, and portable electronic platforms [4,5]. Meanwhile, most high-conductivity shielding materials are dominated by surface reflection, redirecting electromagnetic energy rather than dissipating it. This secondary electromagnetic pollution can interfere with nearby sensitive circuits and runs counter to the "green shielding" concept that prioritizes absorption-dominated attenuation [69]. In addition, conventional shielding materials generally lack intrinsic deformability, self-healing capability, and stimuli-responsiveness, which limits their integration with emerging soft and wearable electronic systems [70,71].

Gel-based materials are well suited to address these limitations. Their tunable network structure and adjustable filler loading allow rational control of impedance matching. Moderate conductivity combined with graded or hierarchical porosity can favor absorption-dominated shielding while avoiding excessive front-surface reflection [69]. These properties, detailed in Section 2, enable conformal integration with curved, dynamic, or biological surfaces. In the following subsections, representative studies are organized by gel type, with each subsection structured around the core design strategies, the role of the gel matrix in enabling shielding performance, and the remaining limitations of each gel category.

#### 3.2.1. Aerogel-Based Electromagnetic Shielding

Aerogel-based EMI shielding materials exploit ultra-low density, high porosity, and large specific surface area to achieve high specific shielding effectiveness (*SSE*, normalized by density) and absorption-dominated shielding via multiple internal reflections within the interconnected porous network [72,73].

To realize absorption-dominated EMI shielding using aerogel materials, tailoring pore architecture is the core design strategy, which works by optimizing impedance matching to minimize front-surface reflection and extending electromagnetic wave propagation to maximize internal energy dissipation. The most direct implementation of this strategy is building a continuous impedance gradient via oriented pore design. As reviewed by Wu et al. [74], absorption-dominated shielding arises from the oriented pore structure of 3D biomimetic graphene/MXene aerogels. The aligned unidirectional channels establish a gradual impedance transition that guides electromagnetic waves into the material interior and enhances internal dissipation via multiple scattering [74]. Beyond unidirectional pore design, hierarchical pore network tuning via fiber stacking offers another effective optimization route. Lan et al. prepared biomass-derived hollow carbon fiber aerogels, where orthogonally layered samples achieved a total SE of 57.3 dB at 10 GHz, nearly double that of parallel-aligned counterparts [75]. The orthogonal stacking of hollow fibers maximizes scattering interfaces and improves impedance matching, which in turn strengthens internal reflection and polarization loss. These design rules are further confirmed in a review by Cheng et al. [43]. They demonstrated that graphene aerogels exhibit higher absorption coefficients than conventional reflection-dominated shielding materials such as MXene films, graphene films, and metal aerogels, as shown in Table 4. This superior absorption performance is primarily attributed to the unique porous structure of aerogels. The interconnected three-dimensional pore networks provide abundant internal reflection sites for incident waves. Meanwhile, the porous framework keeps the conductivity of partially reduced graphene moderate, which reduces impedance mismatch and surface reflection.

**Figure 7:**
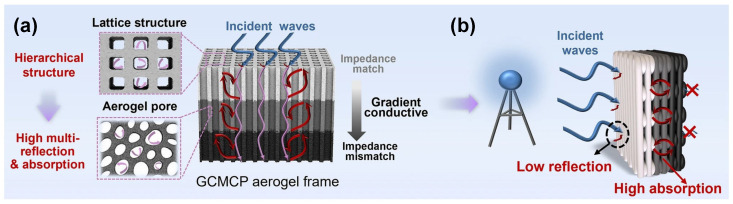
Schematic diagram of the EMI shielding mechanism in gradient-conductive aerogel frames. (**a**) Microscopic wave attenuation inside hierarchical gradient GCMCP aerogel frame with successive impedance matching. (**b**) Macroscopic electromagnetic wave response featuring low surface reflection and high internal absorption (Adapted from Ref. [45]).

Beyond regulating pore architecture, constructing spatial conductivity gradients has emerged as an effective strategy for absorption-dominated EMI shielding in aerogels. Uniformly conductive aerogels often suffer from strong front-surface reflection because the large impedance mismatch prevents electromagnetic waves from entering the porous interior. By contrast, gradient-conductive structures can progressively improve impedance matching between free space and the shielding material, allowing incident waves to penetrate deeper into the aerogel and undergo enhanced attenuation through conductive loss and multiple internal reflections within the porous network. Xue et al. [45] demonstrated this strategy by fabricating MXene/CNT/polyimide aerogel frames with continuously graded conductivity via 3D printing, as shown in Figure 7. The conductivity gradient was achieved by varying the CNT loading across the printed structure, which effectively reduced front-surface reflection while maintaining high shielding performance. The resulting aerogel exhibited an average shielding effectiveness of 68.2 dB together with an ultra-low reflection coefficient of 0.23. In addition, the hierarchical porous architecture prolonged electromagnetic propagation paths inside the aerogel, further enhancing internal scattering and energy dissipation.

In fact, the porous structure of aerogels can simultaneously provide EMI shielding and host other complementary functions in an integrated manner. For example, Liu et al. used the porous interior of cellulose/MXene aerogels to maintain efficient electromagnetic shielding, while introducing a coral-like polyaniline (PANI) skin to enhance interfacial polarization and long-term stability [76]. Beyond structural stability, the porous framework can also enable thermal management functions. Kim et al. designed an asymmetric MXene/TEMPO-oxidized cellulose nanofiber (T-CNF) aerogel supported by an octet-truss (OT) framework, with n-eicosane (EC) as the phase-change material (PCM) to form the OT-MXA-EC composite [77]. The OT framework improves mechanical resilience and stabilizes the composite during phase transitions, addressing the mechanical fragility and limited thermal energy storage capacity that typically limit MXene-based composites.

A key concern for MXene-based aerogel shields is oxidative degradation in humid environments, which gradually reduces electrical conductivity and long-term shielding performance [17,78].

#### 3.2.2. Hydrogel-Based Electromagnetic Shielding

Unlike aerogels that rely primarily on tailored porous structures and conductive fillers for EMI shielding, hydrogels provide a unique attenuation mechanism. Their high water content contributes directly to electromagnetic wave dissipation through dipolar relaxation of water molecules, which complements conductive and interfacial polarization losses from embedded fillers. This synergistic effect allows hydrogels to achieve high-performance broadband EMI shielding at ultra-low filler loadings. Yang et al. [79] fabricated biomimetic MXene/poly(vinyl alcohol) (PVA) hydrogels with aligned honeycomb-like porous structures via ice-templated freezing and salting-out treatment. The aligned pore walls enhanced internal electromagnetic wave reflections. The retained water offered additional dielectric loss. This delivered a shielding effectiveness (SE) of 57 dB in the X-band at only 0.86 vol% MXene loading. Moreover, the SE remained above 50 dB across an ultrabroad frequency range of 8.2–40 GHz. In-situ dehydration experiments further quantitatively verified the critical contribution of water to the overall shielding performance. These experiments confirmed the unique attenuation role of the hydrogel matrix [79].

**Figure 8:**
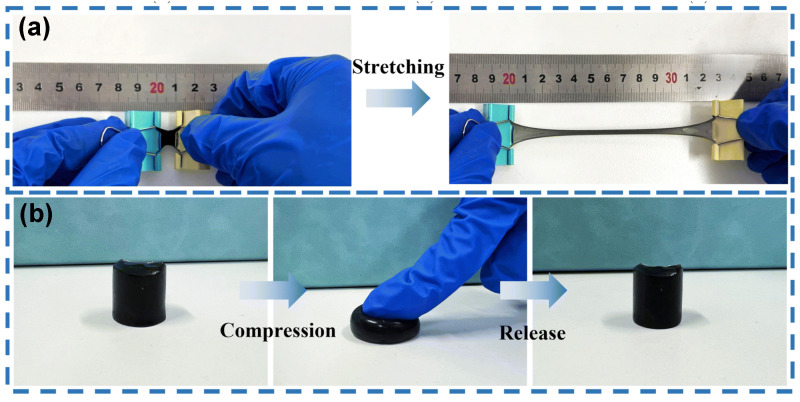
Photographs showing the excellent mechanical deformation performances of dual-network hydrogel. (**a**) Large stretching deformation. (**b**) Compression and full recovery (Adapted from Ref. [80]).

A key advantage of hydrogels over aerogels is their ability to maintain stable EMI shielding under large deformation. For instance, Lian et al. developed a conductive, stretchable, and optically transparent P(AM-co-AA) hydrogel by cross-linking acrylamide (AM) with acrylic acid (AA) [28]. The hydrophilic carboxyl and acyl amino groups in the polymer chains created a favorable hydrogen bonding environment, and the rich polymer network formed at an optimized monomer ratio enhanced both the mechanical properties and the EMI shielding performance. The hydrogel achieved 37 dB SE in the K-band and retained 20 dB SE at 150% strain, owing to the stable ionic conduction pathway in the hydrated polymer network. Similarly, Hao et al. fabricated freeze-cast Fe_3_O_4_/PEDOT:PSS/PVA composite hydrogels with outstanding stretchability up to 904.5% and over 46 dB SE in 8–12.5 GHz, in which the porous structure ensured efficient electromagnetic attenuation under large deformation [47]. Furthermore, Pu et al. proposed a dual-network hydrogel with 850% elongation and 34.5 dB SE, where the robust structure maintained structural integrity and conductive pathways under stretching [80]. The mechanical deformability of this hydrogel is demonstrated by its large stretching and compression recovery behaviors (Figure 8).

The main challenge for hydrogel-based EMI shields is that achieving ultrahigh SE at low filler loading requires careful structural optimization, because excessive filler content compromises intrinsic flexibility and transparency. Advances in biomimetic pore design and graded architectures offer promising routes to resolve this trade-off.

#### 3.2.3. Ionogel-Based Electromagnetic Shielding

Compared with conventional hydrogels, ionogels exhibit superior environmental stability because the non-volatile ionic liquid phase suppresses solvent evaporation and maintains ionic conductivity over a broad temperature range. More importantly for EMI shielding, the ionic liquid phase simultaneously provides ionic conduction and dielectric polarization loss, enabling efficient electromagnetic energy dissipation. Combined with their intrinsic flexibility and mechanical robustness, these features make ionogels promising candidates for flexible, adaptive, and low-reflection EMI shielding systems [29].

**Figure 9:**
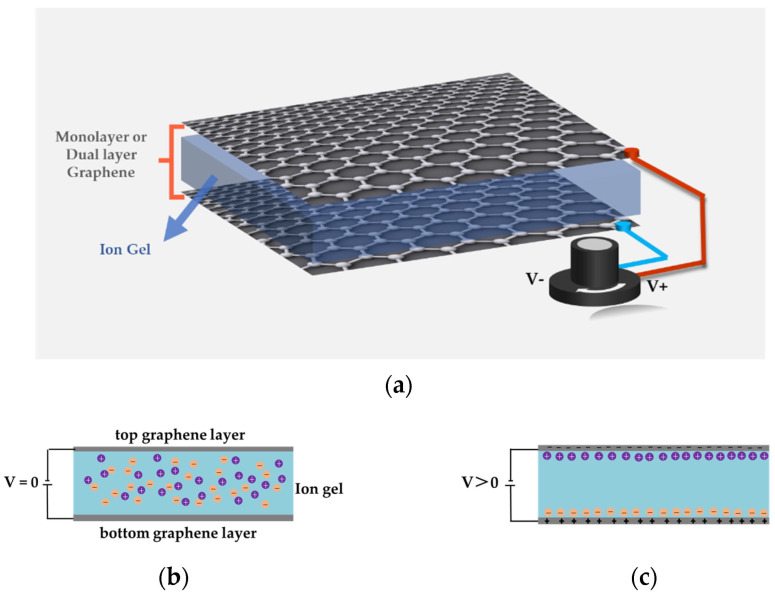
Schematic illustration of the graphene/ionogel/graphene (GIG) sandwich structure and its voltage-controlled tuning mechanism. (**a**) Structure of the GIG device composed of graphene electrodes separated by an ionogel layer. (**b**) Random ion distribution without external bias. (**c**) Formation of electric double layers and charge accumulation at the graphene/ionogel interfaces under applied voltage, enabling tunable electromagnetic shielding performance (Reprinted from Ref. [50]).

A unique advantage of ionogels in EMI shielding is their ability to enable electrically tunable shielding performance. Yao et al. [50] developed a graphene/ionogel/graphene sandwich structure in which the ionogel served as a solid electrolyte, as illustrated in Figure 9. Under an applied voltage, ion migration within the ionogel formed an electric double layer (EDL) at the graphene interface, dynamically modulating the graphene conductivity and therefore the shielding effectiveness (SE). The device achieved more than 10 dB tunable SE across both GHz and THz frequency ranges with second-scale response times, demonstrating the potential of ionogels for smart and adaptive EMI shielding systems.

Ionogels have also been combined with liquid metals to construct flexible shielding materials with high conductivity and mechanical robustness. In these systems, the ionogel not only preserves the softness of the composite, but also stabilizes the liquid metal and prevents leakage. Wang et al. [81] encapsulated liquid-metal-coated textiles within a P(AAm-co-AA) ionogel layer, achieving stable EMI shielding from −18 to 100 °C together with an average SE of 73.0 dB for a three-layer structure. In a subsequent fiber-shaped design, the same group used an ionogel shell to confine a liquid metal core, where the ionogel simultaneously acted as the structural support and impedance matching layer. Three stacked fibers achieved approximately 70 dB shielding effectiveness with an ultra-low reflection coefficient of 0.14 at 10 GHz [82]. These studies highlight the important role of ionogels in flexible, stretchable, and low-reflection EMI shielding devices.

Ionogel-based EMI shielding systems remain less explored than aerogel- and hydrogel-based counterparts, and the voltage-tunable shielding concept has been demonstrated in only a limited number of device configurations [29].

Table 5 summarizes representative gel-based EMI shielding materials, categorized by the three gel systems detailed in this work, highlighting the material composition, the role of the gel matrix, and key shielding performance metrics.

In summary, aerogel-based shields achieve the highest specific shielding effectiveness among gel systems (up to 91 dB in the X-band) through the combined effect of porous impedance matching and multiple internal scattering, with gradient-conductive architectures further reducing the reflection coefficient to values as low as 0.23. Hydrogel shields offer a complementary advantage: their water-mediated dipolar loss enables high SE at ultralow filler loadings (57 dB at 0.86 vol% MXene) while preserving stretchability exceeding 900%. Ionogel shields, though less extensively investigated, enable electrically tunable SE via electric double layer gating with tuning ranges exceeding 10 dB. The primary challenge common to all three systems is long-term performance stability: MXene-based aerogels are susceptible to oxidation in humid environments, hydrogels suffer from dehydration-induced SE degradation, and the leakage behavior of ionogel shields under cyclic mechanical loading remains insufficiently characterized.

### 3.3. Gels in Electromagnetic Wave Absorption

Electromagnetic wave absorption refers to the conversion of incident microwave energy into thermal energy within the absorber material. It is characterized by the reflection loss (RL) measured at the front surface of a metal-backed single-layer sample. Based on transmission-line theory, the value of RL (in dB) is determined by the material’s input impedance ($Z_{in}$) and free-space impedance ($Z_{0}$) via:

(1) $$RL=20\log_{10}\left|\frac{Z_{in}-Z_{0}}{Z_{in}+Z_{0}}\right|$$

A material is considered an effective absorber when RL≤−10 dB, which corresponds to absorbing more than 90% of the incident power. The frequency range satisfying this criterion is defined as the effective absorption bandwidth (EAB) [44]. If $f_{H}$ and $f_{L}$ denote the upper and lower frequency bounds where the reflection loss remains below this threshold, the EAB is mathematically defined as:

(2) $$\mathrm{EAB}=f_{H}-f_{L}\;\left(\mathrm{for}\;RL\leq -10\;\mathrm{dB}\right)$$

Unlike EMI shielding, which tolerates surface reflection as long as transmitted power is minimized, microwave absorption requires simultaneous impedance matching ($Z_{in}\approx Z_{0}$) to allow wave penetration and maximize the EAB, making it inherently more difficult to design [3].

Current microwave absorbers are limited by several critical challenges. Traditional absorbers based on ferrites and metal particles suffer from high density and narrow effective absorption bandwidths. These drawbacks restrict their use on platforms with strict weight constraints, such as aircraft and unmanned aerial vehicles [84]. Carbon-based powder absorbers (e.g., carbon nanotubes, graphene flakes) typically require mixing with polymer matrices at high filler loadings. This often results in poor impedance matching due to excessively high permittivity, which reflects rather than absorbs incident waves [11]. Moreover, conventional absorbers are rigid monoliths that cannot conform to complex curved surfaces or adapt to dynamic mechanical environments [3].

Gel-based materials offer unique structural and compositional advantages for overcoming these limitations, as analyzed in detail in Section 2. The following subsections organize representative studies by gel type, highlighting the design strategies and the role of each gel category in enabling absorption performance.

#### 3.3.1. Aerogel-Based Electromagnetic Wave Absorption

Aerogel-based microwave absorbers exploit ultralow density, high porosity, and large specific surface area to achieve lightweight broadband electromagnetic wave attenuation [11,42]. Their high air volume fraction gives aerogels an effective permittivity close to free space, naturally improving impedance matching and allowing incident electromagnetic waves to enter the material with reduced surface reflection. Meanwhile, the abundant porous interfaces and conductive networks promote multiple attenuation mechanisms, including conduction loss, dipole polarization, interfacial polarization, and multiple internal reflections [19].

Aerogel absorption performance can be directly engineered through pore architecture. Huang et al. [85] fabricated graphene aerogels through a freeze-thaw assembly strategy involving chemical pre-reduction, freeze-thaw cycling, and freeze-drying. By adjusting the pre-reduction time, they obtained tunable cellular structures and demonstrated that the pore size and porosity strongly influence multiple reflections, impedance matching, and attenuation. By optimizing the porous geometry, the aerogel achieved a minimum reflection loss of −61.63 dB and an effective absorption bandwidth of 7.8 GHz at an ultralow filler loading of only 0.74 wt%.

The ultralow density and interconnected conductive pathways of aerogels also enable strong absorption at very low filler loadings. Cai et al. [19] fabricated MXene/CoNi lamellar aerogels via a top-down ice template method, comparing disordered, porous, and lamellar architectures. With a density of only 0.015 g·cm^−3^, the lamellar configuration exhibited the strongest electrical and magnetic coupling effects and superior impedance matching. At only 6 wt% filler loading, the aerogel achieved a minimum reflection loss of −53.87 dB and an effective absorption bandwidth of 6.84 GHz. The aligned porous channels prolonged electromagnetic wave propagation paths and enhanced conduction loss within the lightweight framework.

**Figure 10:**
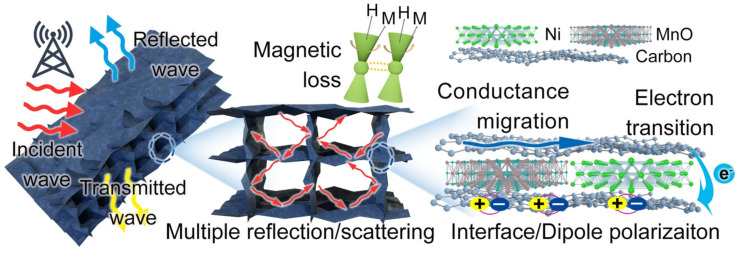
Schematic diagram of electromagnetic wave absorption mechanisms for Ni/MnO nitrogen-doped carbon aerogel (Ni/MnO-CA), including conduction loss, magnetic resonance, interfacial polarization, and multiple reflections (Reprinted from Ref. [42]).

Another important feature of aerogels is their ability to integrate multiple loss mechanisms within a single porous architecture. Wang et al. [42] constructed Ni/MnO nitrogen-doped carbon aerogels in which conductive carbon networks, magnetic nanoparticles, and heterogeneous interfaces simultaneously contributed to conduction loss, magnetic resonance, and interfacial polarization (Figure 10). The resulting aerogel achieved a minimum RL of −64.09 dB and a broad EAB of 7.36 GHz, demonstrating the effectiveness of multi-mechanism synergistic attenuation.

Beyond microwave absorption, aerogels can simultaneously provide thermal insulation and environmental stability for multifunctional stealth applications. Wu et al. [11] developed MXene/C nanofiber aerogels that combined strong microwave absorption performance with excellent thermal insulation capability, highlighting the potential of aerogels for aerospace stealth and thermal protection systems.

A practical concern for aerogel absorbers is the processing cost, because maintaining the high-porosity architecture requires energy-intensive freeze-drying or supercritical drying steps, which limit large-scale production [11,19].

#### 3.3.2. Hydrogel-Based Electromagnetic Wave Absorption

Hydrogel-based microwave absorbers provide a distinctive attenuation mechanism unavailable to aerogels. The high water content within the gel matrix dissipates electromagnetic energy through dipolar relaxation of bound water molecules, adding an absorption channel that supplements the conduction and polarization losses from embedded fillers [13,39]. This unique liquid-phase contribution enables hydrogels to achieve effective microwave absorption at ultra-low filler loadings and provides the basis for frequency-tunable and environmentally adaptive absorption.

A distinctive advantage of hydrogel-based absorbers is their potential for dynamic frequency tunability via mechanical deformation. Tang et al. [39] developed a graphene oxide (GO)/Fe_3_O_4_ hydrogel in a mPEG-Ac/ACMO network. By applying tensile strain (0–40%) to the PDMS-modified hydrogel, the effective absorption bandwidth shifts continuously to higher frequencies, covering up to 87% of the measured band. This on-demand frequency selectivity is difficult to achieve with conventional solid absorbers. The dipolar relaxation of water provides intrinsic dielectric loss, while GO and Fe_3_O_4_ introduce synergistic dielectric-magnetic loss for efficient attenuation [39].

The aqueous gel matrix also provides hydrogel absorbers with inherent flexibility and biocompatibility, enabling applications inaccessible to rigid aerogel or ceramic systems. Long et al. [86] prepared hematite@carbon nanotube/polyacrylamide (PAM) hydrogel composites by dispersing Fe_2_O_3_@CNT hybrid fillers at loadings of 2, 4, and 6 wt% within the polymer network. The hybrid fillers optimized impedance matching and introduced additional dielectric-magnetic loss that complemented the intrinsic dipolar relaxation of bound water. The composite achieved excellent microwave absorption even at low filler loadings, with a minimum reflection loss of −60.96 dB at 15.87 GHz and nearly complete absorption across the Ku band at optimized thicknesses. The hydrogel matrix simultaneously provided mechanical flexibility, biocompatibility, and water-mediated dipolar loss, demonstrating the potential of structure–function integrated hydrogel absorbers [86].

Recent studies further demonstrate that hydrogel absorbers can integrate broadband microwave attenuation with environmental adaptability. Lan et al. [87] developed a PVA/poly(N-isopropylacrylamide) (PNIPAM) interpenetrating-network hydrogel containing magnetic liquid metal particles and LiCl, achieving complete X-band absorption at only 0.9 mm thickness. The highly entangled porous structure promoted multiple scattering and impedance matching, while Li^+^ migration, dipolar relaxation, and interfacial polarization jointly enhanced dielectric loss. Meanwhile, the hygroscopic LiCl and dynamic polymer network enabled rapid moisture recovery and stable absorption performance under thermal and environmental fluctuations, highlighting the potential of environmentally adaptive hydrogel absorbers [87].

A challenge specific to hydrogel absorbers is the impedance mismatch at the air–gel interface introduced by the intrinsically high permittivity of water-rich hydrogels, which must be managed through structural design or gradient composition [13].

#### 3.3.3. Ionogel-Based Electromagnetic Wave Absorption

Ionogels possess inherently high ionic conductivity, negligible vapor pressure, and wide liquid-temperature ranges. These properties directly address the environmental instability of hydrogel absorbers: the non-volatile ionic liquid eliminates evaporation-induced performance degradation, while its low freezing point maintains absorption functionality at sub-zero temperatures. The ionic conduction loss provided by the mobile ions serves as a dominant attenuation mechanism. It can be rationally tuned by selecting ionic liquid species with different viscosities and conductivities [13].

The effectiveness of ionic conduction loss as a microwave attenuation mechanism was demonstrated by Zhao et al. [13], who immobilized the highly conductive ionic liquid 1-ethyl-3-methylimidazolium ethyl sulfate in a polymer network. The resulting ionogel achieved a wide effective absorption bandwidth spanning 10.79–16.38 GHz at a matching thickness of only 2.2 mm, with the absorption performance directly correlated to the ionic conductivity of the immobilized liquid. The negligible vapor pressure of the ionic liquid also addresses the evaporation-induced degradation that typically limits hydrogel-based absorbers, offering long-term environmental stability without the need for encapsulation.

Beyond intrinsic ionic conduction loss, the mechanical deformability of ionogels also enables structural reconfiguration of microwave absorbers. Wu et al. [48] designed a nano-graphite sheet/ionogel composite integrated with 3D-printed dielectric structures, where the compressible ionogel units could reversibly switch between different geometries. The structural transformation significantly improved impedance matching and broadened the effective absorption bandwidth from 9.45 to 13.45 GHz while maintaining excellent wide-angle absorption performance. This work demonstrates that the softness and recoverability of ionogels can provide a practical route toward configurable and mechanically adaptive all-dielectric absorbers [48].

**Figure 11:**
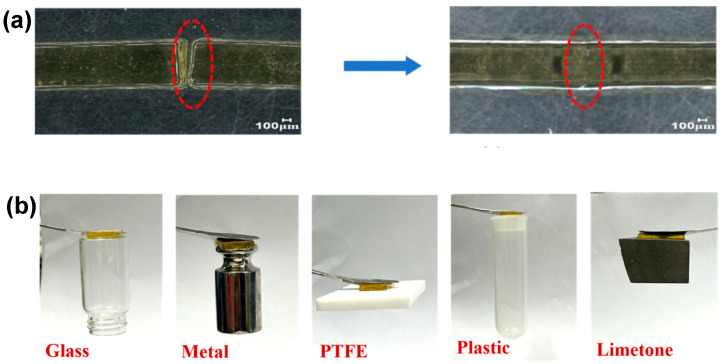
Functional properties of the poly(ionic liquid)-based ionogel absorber. (**a**) Self-healing behavior: optical microscopy images showing a cut sample before and after contact at 60 °C for 20 min; the crack disappears completely, and the healed gel withstands stretching without fracture. (**b**) Conformal adhesion: photographs demonstrating strong adhesion to various substrates including glass, metal, PTFE, ceramic, and limestone (Adapted from Ref. [41]).

Besides improving environmental stability, ionogels also offer practical advantages for deployable microwave absorbers because the ionic liquid–polymer interactions can simultaneously provide softness, adhesion, and dynamic reversibility. Wang et al. [41] developed a poly(ionic liquid)-based ionogel absorber that achieved a maximum RL of −45.7 dB and an EAB of 8.08 GHz while exhibiting self-healing, recyclability, and conformal adhesion to irregular surfaces (Figure 11). These properties allow the absorber to recover from mechanical damage and maintain intimate surface contact during service, overcoming the brittleness and poor adaptability of conventional rigid absorbers.

#### 3.3.4. Other Gel-Based Electromagnetic Wave Absorption

In addition to aerogels, hydrogels, and ionogels, several other gel-type systems have emerged as microwave absorbers, each addressing specific limitations of the three primary categories. Organohydrogels and xerogels represent distinct strategies for expanding the operational envelope of gel-based absorbers.

Organohydrogels, which employ organic solvent/water binary mixtures (e.g., glycerol/water) as the liquid phase, directly overcome the freezing and evaporation instabilities of pure hydrogels while retaining liquid-phase dipolar loss contributions. Zhang et al. fabricated poly(acrylamide-co-acrylic acid) organohydrogels using a glycerol/water mixed solvent, achieving a minimum RL of −33.8 dB at 12.4 GHz with an effective absorption bandwidth covering the entire X-band at a thickness of 2.7 mm [40].

Xerogels, obtained by ambient-pressure drying of wet gels, offer a scalability advantage over aerogels. Medeiros et al. synthesized sustainable carbon xerogels from tannin and achieved a reflection loss of −43.19 dB at 13.79 GHz [88].

Table 6 summarizes representative gel-based electromagnetic wave absorption materials, categorized by the four gel systems discussed in this section, highlighting the material composition, the role of the gel matrix, and key absorption performance metrics.

In summary, aerogel-based absorbers currently achieve the broadest effective absorption bandwidths among gel systems (up to 7.8 GHz at 0.74 wt% filler) and the deepest reflection losses (below −64 dB), with the porous architecture providing simultaneous impedance matching and multiple internal scattering. Hydrogel absorbers offer a unique water-mediated attenuation pathway that enables frequency-tunable absorption through mechanical strain, covering up to 87% of the measured band via tensile deformation. Ionogel absorbers address the environmental instability of hydrogels through non-volatile ionic liquids, with demonstrated EABs exceeding 13 GHz in structurally reconfigurable designs. Organohydrogels and xerogels represent complementary strategies for anti-freezing operation and scalable ambient-pressure fabrication, respectively. The central trade-off across all gel-based absorbers is between performance and practical durability: the highest-performing aerogel absorbers are mechanically fragile and energy-intensive to produce, while more scalable systems generally show reduced porosity and narrower absorption bandwidths.

### 3.4. Gels in Radomes

A radome is a protective enclosure for antenna systems. It shields antennas from environmental damage while allowing electromagnetic waves to pass through with minimal attenuation and distortion [38]. Therefore, radome materials require a low dielectric constant ($\varepsilon_{r}$ close to 1) and low dielectric loss (tanδ), ensuring stable antenna transmission and radiation performance [89].

With the development of hypersonic vehicles and advanced aerospace systems, modern radomes must simultaneously provide broadband electromagnetic transparency, thermal insulation against aerodynamic heating, and sufficient mechanical robustness under harsh environments [20,38]. Conventional materials often show strong trade-offs between these requirements. Ceramics possess excellent thermal stability but relatively high dielectric constants. Polymer composites offer good electromagnetic transparency but poor high-temperature resistance. Aerogel-based materials provide a promising solution because their ultrahigh porosity gives rise to near-unity permittivity, ultralow dielectric loss, and low thermal conductivity simultaneously.

Aerogels are particularly attractive for radome applications because their high air volume fraction produces effective dielectric properties close to free space. This enables excellent electromagnetic transparency over microwave and millimeter-wave bands. Meanwhile, their nanoscale porous structures strongly suppress heat transfer through the Knudsen effect, providing efficient thermal insulation [15,20]. In the following sections, representative gel-based radome materials are reviewed according to their material systems.

#### 3.4.1. Boron Nitride Aerogels for Radomes

Boron nitride (BN) is an intrinsically wave-transparent ceramic with low dielectric loss and excellent thermal stability. Wang et al. [20] fabricated BN nanobelt aerogels with an ultralow density of 18 mg·cm^−3^, dielectric constant of 1.03, and low dielectric loss over 4–18 GHz. The aerogel also exhibited low thermal conductivity and thermal stability up to 1400 °C in inert atmosphere, making it suitable for high-temperature radome thermal protection. Later BN composite aerogels further improved thermal insulation while preserving excellent wave transparency [90].

To improve mechanical robustness, Zhang et al. [49] introduced boron nitride nanosheets into SiO_2_ microfiber aerogels, producing compressible composite aerogels with enhanced thermal insulation and maintained broadband electromagnetic transparency for aircraft radome applications.

#### 3.4.2. Silicon Nitride-Based Aerogels for Radomes

Silicon nitride (Si_3_N_4_) is widely used in high-temperature radomes because of its excellent thermal shock resistance and mechanical strength [38]. However, dense Si_3_N_4_ exhibits a relatively high dielectric constant. Constructing porous aerogel architectures effectively reduces the effective permittivity while preserving high-temperature stability.

Zhang et al. [15] developed Si_3_N_4_@SiO_2_ nanowire aerogels with ultralow density, near-unity dielectric constant (1.02–1.06), ultralow loss tangent, and thermal stability above 1200 °C. The aerogels also showed excellent compressibility and thermal insulation, demonstrating strong potential as lightweight radome thermal protection materials. Tong et al. [21] further designed gradient-pore Si_3_N_4_ nanofiber aerogels using a gas-phase self-assembly strategy with a reactive template, forming a nanoparticle-fiber interpenetrating structure. The porosity gradient suppressed frequency-dependent dielectric variation ($\varepsilon^{\prime }=2.31$–2.39, tanδ<0.08 across 8–18 GHz) while maintaining compression resilience. The aerogel achieved stable dielectric performance and remarkable thermal insulation capability, with a backside temperature reduction of 893 °C under heating conditions.

The primary unresolved challenge for gel-based radomes is the simultaneous optimization of near-unity electromagnetic transparency, high-temperature thermal insulation, and structural integrity across a wide service temperature range, a combination that confronts the inherent trade-offs of highly porous ceramic architectures.

The key properties of these representative aerogels are summarized in Table 7.

In summary, ceramic aerogels represent the leading gel-based radome material platform, with BN aerogels achieving $\varepsilon_{r}\approx 1.03$ and thermal stability to 1400 °C in inert atmosphere and Si_3_N_4_@SiO_2_ nanowire aerogels reaching $\varepsilon_{r}\approx 1.02$–1.06 with stability above 1200 °C. The combination of near-unity permittivity, low dielectric loss, and high-temperature thermal insulation makes aerogels uniquely suited for hypersonic radome applications where conventional ceramics and polymer composites cannot simultaneously satisfy all requirements. Performance limitations center on mechanical robustness and moisture sensitivity: highly porous aerogels remain fragile under aerodynamic loading, and hygroscopic oxide-based systems can absorb moisture that raises the effective permittivity. Balancing electromagnetic transparency, thermal protection, and structural integrity across a wide service temperature range remains the primary design challenge for gel-based radomes.

### 3.5. Gels in Other Electromagnetic Applications

Beyond antennas, electromagnetic shielding, wave absorption, and radomes, gels have also shown growing potential in several emerging electromagnetic applications due to their unique combination of softness, ionic conductivity, tunable dielectric properties, and biocompatibility. Representative examples include soft electromagnetic actuators, energy harvesting devices, THz components, tunable metamaterials, low-dielectric packaging materials, and bioelectronic interfaces.

#### 3.5.1. Magneto-Responsive Gel Actuators

Magneto-responsive gels (ferrogels), formed by incorporating magnetic particles into soft gel matrices, enable flexible electromagnetic actuators that overcome the rigidity and poor conformability of conventional magnetic devices [26]. Their combination of magnetic responsiveness and mechanical softness makes them attractive for soft robotics, wearable systems, and adaptive electromagnetic devices. Representative studies include untethered soft robots based on aligned magnetic nanorods [91] and self-sensing hydrogel actuators capable of simultaneous actuation and tactile feedback [92]. Recent advances in programmable magnetization and 4D printing further expand the functionality and fabrication scalability of ferrogel systems [93]. However, practical applications remain limited by the trade-off between magnetic loading and mechanical flexibility, as well as the long-term stability of magnetic particles in hydrated environments.

#### 3.5.2. Gel Materials for Electromagnetic Energy Harvesting

Gel materials have become important functional components in flexible electromagnetic energy harvesting systems, particularly triboelectric nanogenerators (TENGs) and piezoionic devices. Ionogels are widely used as stretchable and transparent ionic electrodes in TENGs because they avoid the freezing and dehydration problems of hydrogels while maintaining excellent mechanical flexibility [94]. For example, Liao et al. [25] developed a transparent self-healing ionogel TENG that operated stably over a wide temperature range. In addition to serving as electrodes, gels can also act as active triboelectric layers or ion-conductive matrices in piezoionic systems, where pressure-induced ion migration generates electrical signals [95]. Despite these advantages, the relatively low power density and long-term mechanical durability of gel-based harvesters still require improvement.

#### 3.5.3. Gel Materials for Terahertz Wave Devices

Gel materials are used in THz devices because their ionic conduction and dipolar relaxation mechanisms provide tunable THz responses that are difficult to achieve with conventional materials [22]. Xie et al. [22] developed a transparent organohydrogel THz absorber with broadband absorption and high optical transparency, where ionic conduction within the gel acted as the main THz attenuation mechanism. Gel materials also enable actively tunable THz devices. For example, hydrogel swelling can regulate the resonance behavior of THz metasurfaces [96], while ionogel-based electrical double-layer gating has been used for dynamically tunable THz filters and sensors [97]. However, maintaining stable dielectric properties under long-term environmental exposure remains a major challenge for practical THz applications.

#### 3.5.4. Gel Materials for Electromagnetic Metamaterials and Metasurfaces

The stimuli-responsive dielectric and conductive properties of gels make them promising building blocks for flexible and tunable electromagnetic metamaterials. Yang et al. [23] demonstrated ionogel-based all-dielectric metamaterials capable of broadband microwave absorption and low-loss frequency-selective transmission, highlighting the possibility of replacing rigid metallic resonators with transparent and flexible gel systems. In addition, ionogels can function as tunable dielectric layers in active metasurfaces. Yao et al. [24] used an ionogel as the solid electrolyte in a graphene-based metasurface to dynamically tune both absorption frequency and reflection amplitude through electrical double-layer gating. Although gel-based metamaterials offer excellent flexibility and tunability, their relatively high dielectric loss and limited long-term electrical stability still constrain high-frequency applications.

#### 3.5.5. Gel Materials for Low-Dielectric Packaging Applications

Gel materials also show strong potential in electromagnetic packaging applications that require low dielectric constant, low dielectric loss, and high electrical insulation performance. Silicone gels are already widely used in power electronic packaging because of their high dielectric strength and excellent stress-relief capability under thermal cycling [98]. For high-frequency applications, aerogels and sol-gel-derived porous materials provide ultralow dielectric constants close to that of air. Meador et al. [14] developed polyimide aerogels with extremely low dielectric constants for microwave packaging and antenna substrates. Sol-gel-derived low-dielectric fillers and ceramic composites have also been explored for high-frequency circuit substrates [99,100]. Remaining challenges include moisture sensitivity, mechanical fragility, and compatibility with standard semiconductor fabrication processes.

#### 3.5.6. Gel Materials for Bioelectronic Interfaces

Hydrogel materials are increasingly important in bioelectronic interfaces because their soft, ion-conductive, and biocompatible nature enables improved electrical coupling with biological tissues compared with rigid metal electrodes [27]. Zhou et al. [27] developed a conductive hydrogel platform compatible with 3D printing and fabricated monolithic all-hydrogel bioelectronic interfaces for stable electrophysiological recording and stimulation. To improve long-term implantation stability, Zhang et al. [101] designed robust hydrogel coatings that formed continuous interfaces with conventional metal electrodes while maintaining low electrochemical impedance. Stretchable graphene-hydrogel hybrid systems have further enabled conformal wearable and implantable bioelectronics [102]. Although gel-based biointerfaces show excellent promise, long-term in vivo stability and standardized clinical evaluation remain major challenges for practical translation.

Table 8 summarizes representative emerging electromagnetic applications of gel materials.

These emerging applications highlight the versatility of gel materials beyond the four major device categories discussed above. Common to all these applications is the exploitation of gel-specific properties that are difficult to replicate with conventional solid materials: ionic conductivity and electric double layer formation for THz and metamaterial tuning, magnetic compliance for soft actuators, stretchable ionic conduction for energy harvesting, and tissue-like softness for bioelectronic interfaces. These application areas remain at an early stage of development, with most demonstrations limited to individual device-level prototypes. Systematic studies on long-term stability, cycling durability, and scalable fabrication are still largely absent. Bridging these gaps will require moving from proof-of-concept demonstrations to statistically validated performance benchmarks and standardized testing protocols.

## 4. Challenges and Future Perspectives

Although gel-based electromagnetic materials have demonstrated promising performance as reviewed in previous sections, several challenges still limit their practical application.

The first major challenge is long-term stability and reliability. Hydrogels suffer from dehydration and freezing, which gradually degrade conductivity and dielectric performance [28,103]. Aerogels are lightweight and highly porous. However, they are often mechanically fragile and susceptible to pore collapse under repeated deformation [11,17]. In addition, conductive fillers such as MXenes are vulnerable to oxidation in humid environments, leading to conductivity decay and shielding instability [104]. Future work should therefore focus on intrinsically stable gel chemistries and mechanically robust porous structures capable of maintaining electromagnetic performance under complex environmental conditions.

A second major challenge is scalable manufacturing. Many high-performance gel-based electromagnetic materials still rely on complex fabrication methods such as supercritical drying or long freeze-drying processes. These methods are energy-intensive and difficult to scale for industrial production [11,19]. Although techniques such as ambient-pressure drying and additive manufacturing have shown promising potential, challenges related to production efficiency, structural uniformity, and cost control remain unresolved. Future research should therefore focus on scalable and low-cost fabrication strategies compatible with continuous manufacturing and large-area device integration.

Another important issue is device-level integration. In practical applications, electromagnetic behavior is strongly coupled with mechanical deformation, thermal management, and environmental exposure. However, most current studies still focus on material-level performance. For wearable and implantable devices, additional concerns such as biocompatibility, thermal safety, and long-term operational stability also require systematic investigation [27].

Finally, the design of gel-based electromagnetic materials still depends heavily on empirical optimization. Because gels are inherently multi-physics systems involving coupled electromagnetic, mechanical, thermal, and transport processes, predictive design remains difficult. Future research should combine multi-physics simulation with data-driven and machine-learning-assisted approaches to accelerate material optimization and guide the design of gel systems for specific electromagnetic applications [105].

## 5. Conclusions

This review has provided a systematic examination of gel-based materials for electromagnetic devices, spanning hydrogels, aerogels, ionogels, organohydrogels, and xerogels across antennas, EMI shielding, microwave absorption, radomes, and emerging applications. By adopting a unified structure–property–function framework, we have shown how the network architecture, dispersion medium, and filler composition of different gel types govern their electromagnetic response and, in turn, their suitability for specific device functions. A central insight emerging from this cross-application comparison is that each gel category occupies a distinct region in the multidimensional design space defined by electromagnetic performance, mechanical deformability, and environmental stability. Hydrogels excel in permittivity-driven miniaturization and stretchability but are constrained by dehydration and freezing. Aerogels provide unmatched impedance matching and lightweight shielding at the cost of mechanical fragility and processing expense. Ionogels offer wide-temperature ionic conductivity and electrical tunability but face challenges of material cost and potential leakage. Organohydrogels and xerogels represent emerging strategies that partially address the limitations of the primary gel types while introducing their own trade-offs.

The field now stands at a transition point: material-level electromagnetic performance has been convincingly demonstrated across a sufficiently broad range of gel systems and device functions to warrant a concerted effort toward addressing the translational challenges identified in Section 4. Environmental stabilization, mechanical reliability under operational conditions, standardized evaluation protocols, scalable manufacturing routes, device-level integration and safety assessment, and predictive multi-physics design tools collectively define the research agenda that will determine whether gel-based electromagnetic materials fulfill their considerable promise in practical technologies. Progress on these fronts, pursued through interdisciplinary collaboration among materials chemists, electromagnetic engineers, mechanical engineers, and data scientists, will be essential for advancing gel-based electromagnetic materials from laboratory demonstrations to enabling components of next-generation flexible, wearable, and intelligent electromagnetic systems.

## Figures and Tables

**Table 1:** Functional fillers for tailoring electromagnetic properties of gels.

Category	Representative Materials	Primary EM Function	Enhanced Properties	Ref.
Conductive	MXene, carbon nanotubes, graphene, silver nanowires, liquid metal (EGaIn)	Electron transport and conduction loss	Conductivity, $\varepsilon^{\prime \prime }$, shielding effectiveness	[19,45,46]
Magnetic	Fe_3_O_4_, CoNi alloy, nickel, ferrite (NiFe_2_O_4_)	Magnetic resonance and eddy current loss	$\mu^{\prime \prime }$, magnetic loss tangent, absorption bandwidth broadening	[39,42,47]
Dielectric ceramic	BaTiO_3_, TiO_2_, SiO_2_ nanoparticles	Dipolar and interfacial polarization	$\varepsilon^{\prime }$ and $\varepsilon^{\prime \prime }$ tunability, impedance matching	[48,49]
Structural ceramic	Boron nitride, Si_3_N_4_, SiO_2_ nanofibers	Wave transparency and thermal insulation	Low $\varepsilon_{r}$, low tanδ, thermal stability	[15,20,21]
Ionic liquid	[EMI][ES], imidazolium-based ILs	Ionic conduction and electric double layer tuning	Ionic conductivity, wide-temperature stability, voltage-tunable response	[13,29,50]

**Table 2:** Comparison of different gel types for electromagnetic applications.

Gel Type	Key Structure	Main EM Parameter	Main Loss/Function	Best-Fit Devices	Main Limitation
Hydrogel	Water-rich polymer network	High $\varepsilon_{r}$, ionic conductivity	Water dipolar relaxation, ionic conduction	Miniaturized antennas, wearable EMI shields	Dehydration, freezing
Aerogel	Porous gas-filled network	Low $\varepsilon_{r}$, low density	Impedance matching, multiple scattering	Radomes, lightweight absorbers, shielding	Brittleness, drying cost
Ionogel	Ionic liquid in network	Stable ionic conductivity	Ionic conduction, EDL tuning	Tunable antennas, adaptive shielding	Cost, leakage, stability
Organohydrogel	Organic/water binary solvent	Moderated $\varepsilon_{r}$, anti-freezing	Dipolar and ionic loss	Wearable absorbers and shields	Solvent compatibility
Xerogel/Cryogel	Dried porous network	Scalable porosity	Scattering, conduction loss	Absorbers, shields	Pore collapse, weakness

**Table 3:** Representative gel-based antennas and their performance.

Gel Material System	Antenna Architecture	Core Function of Gel	Operating Frequency	Key Performance	Ref.
Aerogel-Based Antennas					
PI aerogel	Microstrip patch antenna	Ultra-low-$\varepsilon_{r}$ substrate ($\varepsilon_{r}\approx 1.16$)	2.6 GHz	Wider BW and higher gain than commercial substrates; ultralight	[14]
PI aerogel (filled waveguide)	Ka-band slotted waveguide array	Ultra-low-$\varepsilon_{r}$ filling for mass reduction	Ka-band (37.5 GHz)	Gain 9 dBi; loss 5.16 dB/m	[59]
SiO_2_ aerogel	C-band circular patch	Ultra-low-$\varepsilon_{r}$ substrate	7.2 GHz	1.5 dB gain improvement; 88% BW improvement; 68.5% weight reduction versus RT5880	[60]
High-temperature aerogel	UWB composite DRA	Thermal insulation + dielectric loading	4.8–10.4 GHz	73.7% BW; 9.2 dBi gain; stable at 1000 °C for 1280 s	[61]
Hydrogel-Based Antennas					
PAAm hydrogel + liquid metal	Stretchable dipole	High-$\varepsilon_{r}$ substrate ($\varepsilon_{r}\approx 79$)	927.5 MHz	Size reduced by 50%; strain-tunable 770–927 MHz	[16]
pH-sensitive hydrogel	Integrated slot antenna–pH sensor	Stimuli-responsive sensing layer	21.7 GHz	Passive wireless; 160 MHz shift over pH 5–12	[55]
Silver-loaded photocurable hydrogel	3D-printed NFC/RFID antenna	Conductive radiator (387 S/cm)	NFC/UHF	Stable at 100% strain for ≥30 days; eye-motion sensing	[46]
Ionogel-Based Antennas					
Ionogel gate dielectric	X-band printed phased array	High-capacitance gate dielectric	10 GHz	Beam steering 0°–22.15°; fully printed; flexible	[65]
Ionogel + PDMS	Dual-band flexible microstrip antenna	Tunable dielectric layer	S-band/C-band	$S_{11}<-30$ dB; mechanical dual-band tuning	[66]
Ionogel voltage-tunable	Electrically reconfigurable microstrip antenna	Variable capacitor via DC-bias permittivity tuning	2.4 GHz	Continuous frequency shift 0.1 GHz (0–5.5 V); compensates bending drift; compact	[67]

Abbreviations: BW = bandwidth, DRA = dielectric resonator antenna, UWB = ultra-wideband, NFC = near-field communication, UHF = ultra-high frequency, PAAm = polyacrylamide, PI = polyimide, PDMS = polydimethylsiloxane.

**Table 4:** Comparison of shielding mechanism parameters between graphene composite aerogels and typical reflection-dominated materials (Data extracted from Ref. [43]).

Material Type	Absorption Coefficient (A)	Reflection Coefficient (R)	A/R Ratio
Graphene composite aerogels	0.55–0.82	0.10–0.44	1.24–8.00
MXene aerogels	0–0.2	0.8–1.0	<0.25
MXene films	0.044–0.2	0.8–0.955	0.046–0.25
Graphene films	0–0.1	0.9–1.0	<0.11
Metal aerogels	0–0.15	0.85–0.939	<0.67

**Table 5:** Representative gel-based materials for EMI shielding.

Material System	Core Function of Gel	SE (dB)	Tested Band	Key Performance	Ref.
Aerogel-Based Shielding					
3D-printed gradient MXene/CNT/PI	Graded porous impedance matching	68.2	X-band	R=0.23	[45]
Heterolayered carbonized MXene/PI	Anisotropic porosity with conductivity gradient	91.0/66.2	X-band/THz	X-band: R=0.40; THz: R=0.33; infrared stealth	[83]
Hierarchical cellulose/MXene/PANI film	Open porosity for lightweight design	62.3	X-band	SSE/*t* = 35,600 dB cm^2^ g^−1^	[76]
Biomass-derived hollow C fiber	Orthogonal alignment maximizes scattering	57.3	10 GHz	Orthogonal SE is double that of parallel	[75]
Hydrogel-Based Shielding					
Biomimetic MXene/PVA hydrogel	Water dipolar loss + honeycomb pores	57.0	X-band	ultralow 0.86 vol% MXene	[79]
Transparent P(AM-co-AA) hydrogel	High transmittance; stretchability	37.0	18–26.5 GHz	>80% transmittance; retains 20 dB SE at 150% strain	[28]
Fe_3__3_O_4_/PEDOT:PSS/PVA hydrogel	Freeze-cast pores; stretchable	46.0	8–12.5 GHz	904.5% stretchability	[47]
Ionogel-Based Shielding					
Graphene/ion gel/graphene	EDL gating for voltage-tunable SE	Tunable	GHz + THz	Tunable SE range > 10 dB	[50]
LM-coated textile/ionogel (×3)	Ionogel stabilizes LM interface	73.0	2–18 GHz	Stable from −18 to 100 °C	[81]
LM–ionogel core–shell fiber (×3)	Stretch-recovery enhances SE	70.0	2–18 GHz	R=0.14	[82]

Abbreviations: SE = shielding effectiveness, SSE/*t* = specific shielding effectiveness per unit thickness, LM = liquid metal, EDL = electrical double layer, R = reflection coefficient.

**Table 6:** Representative gel-based materials for electromagnetic wave absorption.

Material System	Function of Gel	RL_min_ (dB)	EAB (GHz)	Key Performance	Ref.
Aerogel-Based Absorption					
Graphene cellular aerogel	Impedance matching via pore geometry	−61.63	7.8	Ultralow filler loading (0.74 wt%)	[85]
MXene/CoNi lamellar aerogel	Ultralow-density conduction loss	−53.87	6.84	ultralow density of 0.015 g·cm^−3^	[19]
Ni/MnO ice-templated aerogel	Magnetic-dielectric synergy	−64.09	7.36	Specific RL = −253.32 dB/mm; radar/infrared stealth	[42]
MXene/C nanofiber aerogel	Absorption + thermal insulation	−53.02	5.3	Thermal insulation performs well (>30 °C cooling)	[11]
Hydrogel-Based Absorption					
GO/Fe_3_O_4_ hydrogel	Frequency tunability via tensile strain (0–40%)	−62.97	5.89	Frequency tunable up to 87% of band	[39]
Fe_2_O_3_@CNT/PAM hydrogel	Flexibility; biocompatibility; dipolar loss	−60.96	3.4	Ku-band absorption at low filler loading	[86]
PVA/PNIPAM@MLM-LiCl hydrogel	Environmental adaptability; dipolar/interfacial polarization	–	X-band	Full X-band absorption at 0.9 mm	[87]
Ionogel-Based Absorption					
[EMI][ES] polymer ionogel	Ionic conduction loss; non-volatile	–	5.59	2.2 mm thickness	[13]
Nano-graphite sheet/ionogel absorber	Structural reconfigurability; ionic conduction loss	−50	13.45	Configurable bandwidth: 9.45–13.45 GHz	[48]
PIL-based UV-cured ionogel	Self-healing; adhesive; recyclable	−45.7	8.08	Conformal adhesion	[41]
Other Gel-Based Absorption					
P(AM-co-AA) glycerol/water organohydrogel	Anti-freezing; dipolar polarization loss	−33.8	X-band	2.7 mm thickness	[40]
Tannin carbon xerogel/CNT composite	Ambient drying; biosourced	−43.19	–	Ku-band absorption at 13.79 GHz; sustainable	[88]

Abbreviations: RL_min_ = minimum reflection loss, EAB = effective absorption bandwidth, PAM = polyacrylamide, PIL = poly(ionic liquid), CNT = carbon nanotube, [EMI][ES] = 1-ethyl-3-methylimidazolium ethyl sulfate.

**Table 7:** Representative gel-based materials for radome applications.

Material System	Role of Gel Structure	$\varepsilon_{r}$/tanδ	Representative Performance	Ref.
Boron Nitride Aerogels				
BN nanobelt aerogel	Wave-transparent thermal insulation	1.03/0.016	Stable to 1400 °C with low thermal conductivity	[20]
BN composite aerogel	Enhanced thermal insulation with preserved transparency	Near-unity/low	Improved thermal protection for radomes	[90]
SiO_2_/BNNS composite aerogel	Compressible wave-transparent thermal insulation	2.75–2.83/$<6.25\times 10^{-3}$	Improved mechanical resilience and thermal insulation	[49]
Silicon Nitride-Based Aerogels				
Si_3_N_4_@SiO_2_ nanowire aerogel	Near-unity permittivity with thermal insulation	1.02–1.06/ultra-low	Compressible and stable above 1200 °C	[15]
Gradient-pore Si_3_N_4_ aerogel	Integrated EM transparency and thermal protection	2.31–2.39/<0.08	Excellent thermal insulation and compression resilience	[21]

Abbreviations: BNNS = boron nitride nanosheets.

**Table 8:** Representative emerging electromagnetic applications of gel materials.

Application	Role of Gels	Representative Performance	Ref.
Magneto-Responsive Actuators	Soft magnetic matrix for flexible actuation and programmable deformation	Untethered soft robotic motion, self-sensing actuation, and stiffness-tunable manipulators	[91,92,93]
Energy Harvesting	Stretchable ionic electrodes and ion-conductive matrices for TENGs and piezoionic devices	Self-healing transparent TENGs, wide-temperature operation, and bio-compatible mechanical-to-electrical conversion	[25,94,95]
THz Wave Devices	THz absorption and tunable resonance enabled by ionic conduction and gel swelling	Broadband transparent THz absorbers and electrically tunable THz filters	[22,96,97]
Metamaterials and Metasurfaces	Flexible dielectric and tunable layers for active electromagnetic wave regulation	Broadband microwave absorption and electrically tunable metasurface response	[23,24]
Low-Dielectric Packaging	Electrical insulation and ultralow-dielectric packaging materials for high-frequency devices	Stable silicone gel insulation and ultralow-$\varepsilon_{r}$ aerogel packaging substrates	[14,98,99]
Bioelectronic Interfaces	Soft ion-conductive interfaces for electrophysiological recording and stimulation	Stretchable all-hydrogel electrodes and long-term stable neural interfaces	[27,101,102]

Abbreviations: THz = terahertz, TENG = triboelectric nanogenerator.

## Data Availability

No new data were created or analyzed in this study.
